# Early characteristics of fulminant myocarditis vs non-fulminant myocarditis: A meta-analysis

**DOI:** 10.1097/MD.0000000000014697

**Published:** 2019-02-22

**Authors:** Zicheng Wang, Yanwei Wang, Haiyan Lin, Shengjie Wang, Xianlei Cai, Da Gao

**Affiliations:** a Department of Cardiology; b Department of General surgery, Ningbo Medical Center Lihuili Hospital, Zhejiang, China.

**Keywords:** early characteristics, meta-analysis, myocarditis

## Abstract

**Background:**

Fulminant myocarditis (FM) is a sub-category myocarditis. Its primary characteristic is a rapidly progressive clinical course that necessitates hemodynamic support. FM can be difficult to predict at the onset of myocarditis. The aim of this meta-analysis was to identify the early characteristics in FM compared to those of non-fulminant myocarditis (NFM).

**Methods:**

We searched the databases of MEDLINE, EMBASE, CENTRAL, for studies comparing FM with acute NFM from January 1, 2000 to June 1, 2018. The baseline variables were compared in each study. Mean differences (MD) and relative ratios (RR) were calculated.

**Results:**

Seven studies (158 FM patients and 388 NFM patients) were included in the analysis. The FM group had significantly lower systolic blood pressure (SBP), higher creatine kinase (CK), wider QRS duration, lower left ventricular ejection fraction (LVEF), thicker left ventricular posterior wall diameter (LVPWd), higher incidence of ST depression, ventricular tachycardia/ventricular fibrillation (Vt/Vf) and syncope, less incidence of chest pain than the NFM groups. There was no difference in terms of heart rate (HR), c-reactive protein (CRP), fever, dyspnea, white blood cells (WBC), atrioventricular block (AVB), Q waves, ST elevation, interventricular septum diameter (IVSd), or end-diastolic left ventricular diameter (LVEDd) between FM and NFM.

**Conclusion:**

We found that the lower SBP, higher CK, wider QRS duration, lower LVEF, thicker LVPWd, higher incidence of ST depression, Vt/Vf and syncope as well as lower incidence of chest pain were early characteristics of FM.

## 1 Introduction

Myocarditis is an inflammatory disease of the myocardium.^[1]^ It is usually caused by common viral infections which may lead to direct myocardial injury or virally-mediated immune responses.^[2]^ In addition, non-viral infections and various medications as well as systemic autoimmune diseases also can cause myocarditis.^[3]^ Myocarditis can be categorized as non-fulminant or fulminant based on a synthesis of clinical, echocardiographic, histological, and hemodynamic findings.^[4]^ Fulminant myocarditis (FM) was defined as an acute illness that may develop fatal ventricular arrhythmia, rapid and severe hemodynamic instability, cardiogenic shock with multiple organ dysfunction syndrome, and sudden death, usually requiring immediate mechanical circulatory support (MCS) immediately,^[5]^ including extracorporeal membrane oxygenation, intra-aortic balloon pump, or ventricular assist devices.

In the past, patients with FM often died of sudden cardiac arrest or severe heart failure soon after onset. With advances in mechanical circulatory support, even severe cases progressing to cardiac arrest can survive through the acute phase.^[6]^ Nevertheless, at the onset of acute myocarditis, fulminant cases can be difficult to predict.^[7]^ Acute myocarditis has a broad spectrum of clinical presentations that vary from minor symptoms, including chest pain and palpitations associated with mild sinus tachycardia to severe heart failure and life-threatening arrhythmias.^[8]^ Early recognition of the patients at high risk of progression to FM is necessary. The survival rate of patients can be significantly improved according to earlier implantation of MCS devices.^[9]^

Recent reports showed that right heart catheterization, serological biomarkers (such as interleukin-10), viral genome examination, and cardiac MRI can predict the prognosis of acute myocarditis.^[10–13]^ However, these tests require substantial amounts of time, making them unwieldy for the stratification of severity of acute myocarditis, especially in its early stages. In the most severe cases of FM, hemodynamic collapse occurs early during hospitalization, sometimes even within a few hours. Early characteristics of FM, therefore, should be detected easily and efficiently. The primary objective of this meta-analysis was to identify the early clinical signs or laboratory findings in FM and NFM.

## 2 Materials and methods

Our meta-analysis was generated according to the Preferred Reporting Items for Systematic Reviews and Meta-Analyses (PRISMA) statement guidelines and was registered at International Prospective Register of Systematic Reviews (number CRD42018100669).^[14]^

A systematic electronic search was performed on PubMed, EMBASE, and CENTRAL (Cochrane Central Registry of controlled trials) from January 1, 2000 to June 1, 2018. Inclusion was restricted to publications in English. Keywords used included the following: “fulminant myocarditis” and “myocarditis”. There was no restriction as to the type of study. The references of relevant studies as well as reviews, editorials, letters, and related conference abstracts were also searched. Eligible studies had to be published as full-length articles in peer-reviewed journals. Inclusion criteria for study selection included comparative studies comparing the early characteristics of FM with NFM. Eligible studies had to report data of characteristics such as clinical presentations, biochemical markers, ECG findings, and echocardiographic features. Only studies with more than 10 patients in each group were included. Reports of pediatric patients were excluded. An ethics committee or institutional review board was not applicable, because the data collected were all from the database.

When data were reported from overlapping study samples (e.g., several publications from the same group), the most recent study or one with the highest number of patients was included in the analysis. Single case reports and editorials were not included. We extracted data from selected studies using a standardized, pilot-tested extraction template.

The endpoints of the analysis were as follows:

1. Vital signs and clinical presentations (systolic blood pressure (SBP), heart rate (HR), chest pain, fever, syncope, dyspnea);
2. Biochemical markers (white blood cells (WBC), C-reactive protein (CRP), creatine kinase (CK));
3. ECG findings (ST-segment elevation; abnormal Q waves, ST-segment depression, ventricular tachycardia/ventricular fibrillation (Vt/Vf), atrioventricular block (AVB), QRS width);
4. Echocardiographic features (intraventricular septum diameter (IVSd), left ventricular posterior wall diameter (LVPWd), end-diastolic left ventricular diameter (LVEDd), left ventricular ejection fraction (LVEF)).

All parameters were defined according to the study definition.

Two investigators (Wang Zicheng and Wang Shengjie) independently assessed reports for eligibility at title and/or at abstract level, with disagreements resolved by a third reviewer (Cai Xianlei). Studies that met inclusion criteria were selected for further analysis. The risk of bias was evaluated by the same 2 reviewer authors, in accordance with the Cochrane Collaboration methods.

Meta-analysis was performed using Review Manager 5.3 statistical software. Reported event frequencies were used to calculate risk ratio (RR) with 95% confidence intervals (CI). Heterogeneity of the trial results was quantified with the Chi^2^ heterogeneity statistic, and inconsistency was assessed by means of *I*^2^. Results were reported as the *P* value of the Chi^2^ test (*P* < .05 for heterogeneous results) and percent of the *I*^2^. Interpretation of the latter was made by assigning attributes of low, moderate, and high in case of 0 to 25%, 50 to 75%, and more than 75%, respectively. We used a random effects or a fixed effects model based on associated heterogeneity. The random effects model resulted in wider confidence intervals and provided more conservative and robust results; it was used when *I*^2^ > 50%. To study the relevance of publication bias, funnel plots were constructed to graph trial results against their precision.

## 3 Results

### 3.1 Search results

**Figure 1:**
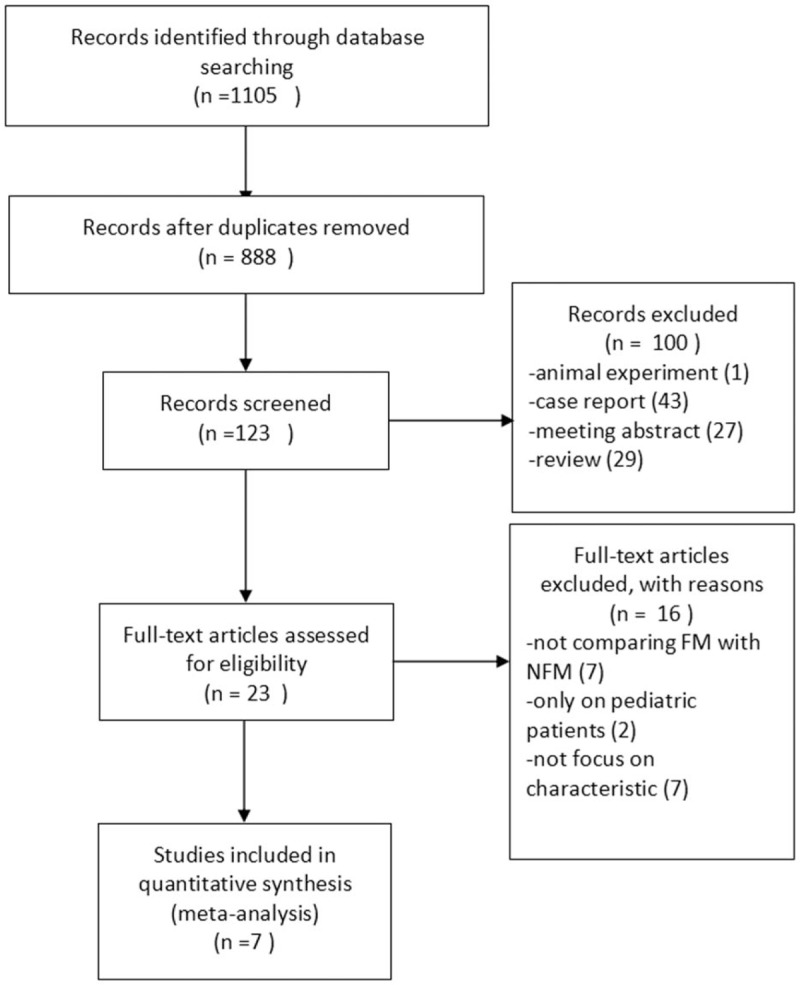
Flowchart of studies from search to inclusion.

Figure 1 summarizes the process of identifying eligible studies. The search yielded 1105 relevant articles. After removal of duplicate articles and duplicate titles, 123 of records were screened for potential eligibility. After removing records related to animal experiments, case reports and reviews, 23 full-text articles were assessed for eligibility. All articles that did not report on comparing characteristics between FM with NFM and those that reported only pediatric patients were removed. According to the inclusion and exclusion criteria for entry into the study, we finally included a total of 7 studies^[15–21]^ in the meta-analysis.

Table 1 presented a description of the 7 papers included in the meta-analysis, including the sample size, publication period, age, gender, and important findings. All papers were published from 2000 to 2018. The studies represented a total of 546 patients. The lowest number of included patients with FM was 9, whereas the largest sample included 55 patients with fulminant myocarditis. One trial^[15]^ evaluated clinical and electrocardiographic characteristics in patients with fulminant myocarditis. Patients in this study were divided into 3 groups; pericarditis as the control, and acute myocarditis, and the FM groups. Biochemical markers and electrocardiogram on admission were then retrospectively analyzed among the 3 groups. In 6 trials,^[15–21]^ patients were divided into fulminant and non-fulminant groups. Clinical data, including clinical presentations, biochemical markers, ECG, and echocardiographic features at admission were compared. Seven trials^[15–21]^ were retrospective analyses. Two trials^[17,18]^ were from Taiwan, 3^[15,16,20]^ were from Japan, 1^[19]^ was from China and one^[21]^ was from Italy. Five trials^[15,17–20]^ included only adults (>16 years old), and in another 2 trials,^[16,21]^ ages were not reported.

**Table 1:**
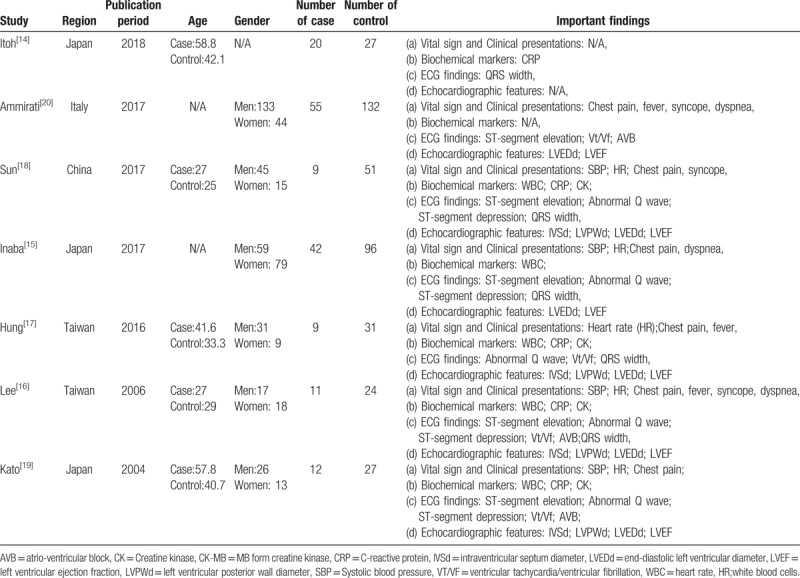
Characteristics of included studies.

### 3.2 Vital signs and clinical presentations

**Figure 2:**
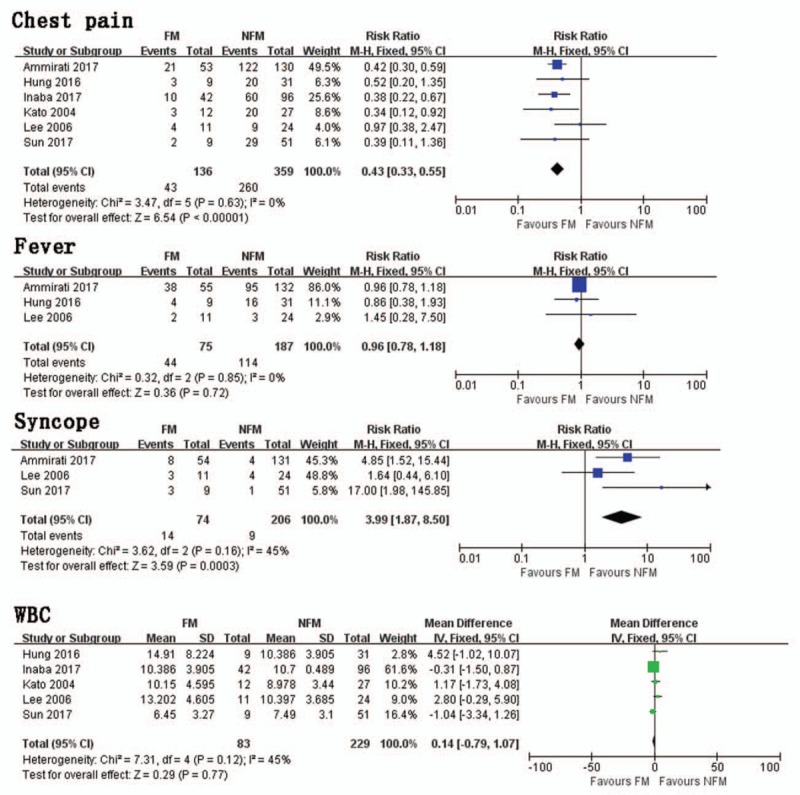
Chest pain. Risk ratio between FM and NFM; Fever. Risk ratio between FM and NFM; Syncope. Risk ratio between FM and NFM; WBC. Mean difference between FM and NFM. FM = fulminant myocarditis, NFM = non-fulminant myocarditis, WBC = white blood cells.

**Figure 3:**
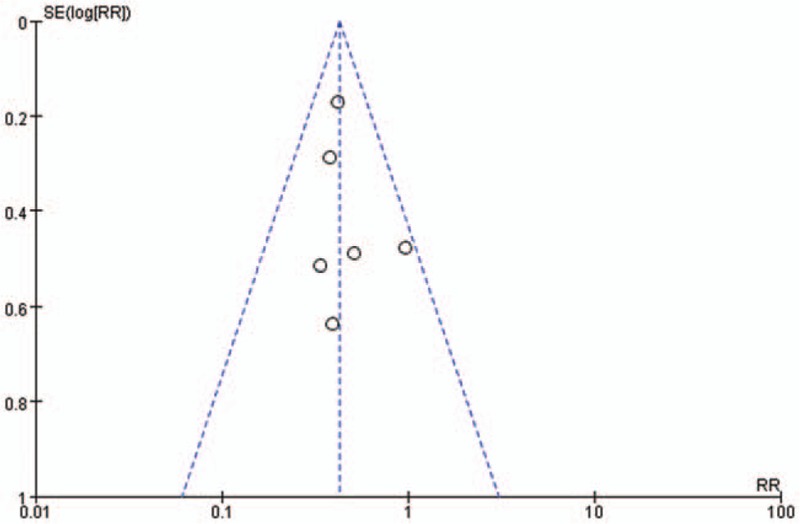
Funnel plot of included studies.

Six studies investigated the occurrence of chest pain between FM and NFM. Results of this pooled analysis indicated that FM group had significantly fewer chest pain presentations than NFM group (RR = 0.43, 95% CI 0.33, 0.55; *I*^2^ = 0, *P* = .63) (Fig. 2). A funnel plot was included (Fig. 3). In a pooled analysis of 3 studies, there was no significant difference in fever between FM and NFM groups (RR = 0.96, 95% CI 0.78,1.18, *I*^2^ = 0, *P* = .85) (Fig. 2). Three trials studied the occurrence of syncope between FM and NFM. The FM group had significantly more syncope presentations than NFM group (RR = 3.99, 95% CI 1.87, 8.50; *I*^2^ = 45%, *P* = .16) (Fig. 2).

Pooled data from 4 studies studied SBP in FM and NFM groups. FM group had significantly lower SBP than NFM group (MD = −14.29, 95% CI −25.87, −2.78, *I*^2^ = 85%, *P* = .0002). Five trials investigated differences in HR. There was no significant difference in HR between FM and NFM groups (MD = 16.30, 95% CI −4.01, 36.6, *I*^2^ = 89%, *P* < .00001). In a pooled analysis of 3 trials, there was no significant difference in dyspnea between FM and NFM groups (RR = 2.28, 95% CI 0.60, 8.69; *I*^2^ = 95%, *P* < .00001).

### 3.3 Biochemical markers

In a pooled analysis of 5 trials, there was no significant difference in WBC between FM and NFM groups (MD = 0.14, 95% CI −0.79, 1.07, *I*^2^ = 45%, *P* = .12) (Fig. 2).

Pooled data from 3 trials evaluated CK value in FM and NFM groups. FM group had significantly higher CK than NFM group (MD = 541.73 95% CI 40.27, 1043.18; *I*^2^ = 53%, *P* = .12). Six trials showed that there was no significant difference in CRP between FM and NFM groups (MD = 1.50, 95% CI −3.19, 6.20; *I*^2^ = 71%, *P* = .02).

### 3.4 ECG findings

**Figure 4:**
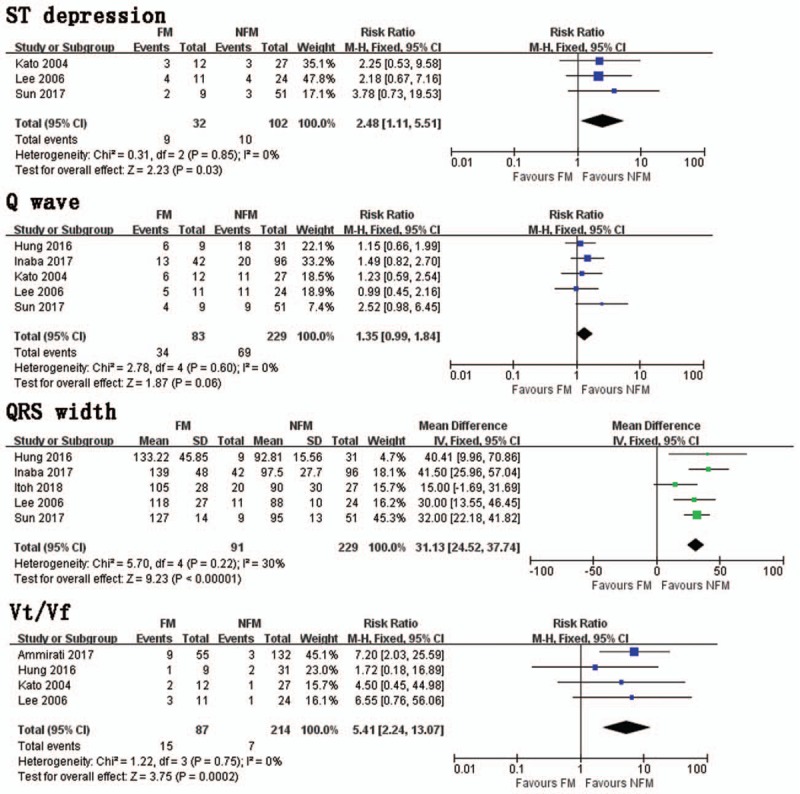
ST depression. Risk ratio between FM and NFM; *Q* wave. Risk ratio between FM and NFM; QRS width. Mean difference between FM and NFM; Vt/Vf. Risk ratio between FM and NFM. FM = fulminant myocarditis, NFM = non-fulminant myocarditis, Vt/Vf = ventricular tachycardia/ventricular fibrillation.

In a pooled analysis of 3 trials, FM group had significantly more ST segment depression presentations than NFM group (RR = 2.48, 95% CI 1.11, 5.51; *I*^2^ = 0%, *P* = .60) (Fig. 4). Pooled data from 5 trials indicated that there was no significant difference in Q waves between FM and NFM groups (RR = 1.35, 95% CI 0.99, 1.84; *I*^2^ = 0%, *P* = .60) (Fig. 4). Five studies investigated QRS width in FM and NFM groups. The FM group had more significant extension of QRS duration than NFM groups (MD = 31.13, 95% CI 24.52, 37.74; *I*^2^ = 30%, *P* = .22) (Fig. 4). Four trials studied the occurrence of Vt/Vf presentations between FM and NFM groups. The FM group had significantly more Vt/Vf presentations than NFM group (RR = 5.41, 95% CI 2.24, 13.07; *I*^2^ = 0%, *P* = .75) (Fig. 4).

Three trials studied the occurrence of AVB and indicated that there was no significant difference in *Q* waves between FM and NFM groups (RR = 1.79, 95% CI 0.61, 5.28; *I*^2^ = 62%, *P* = .07). In a pooled analysis of 5 trials, statistical differences could not be found in ST segment elevation presentations between the 2 group (RR = 0.92, 95% CI 0.60, 1.41; *I*^2^ = 73%, *P* = .005).

### 3.5 Echocardiographic features

**Figure 5:**
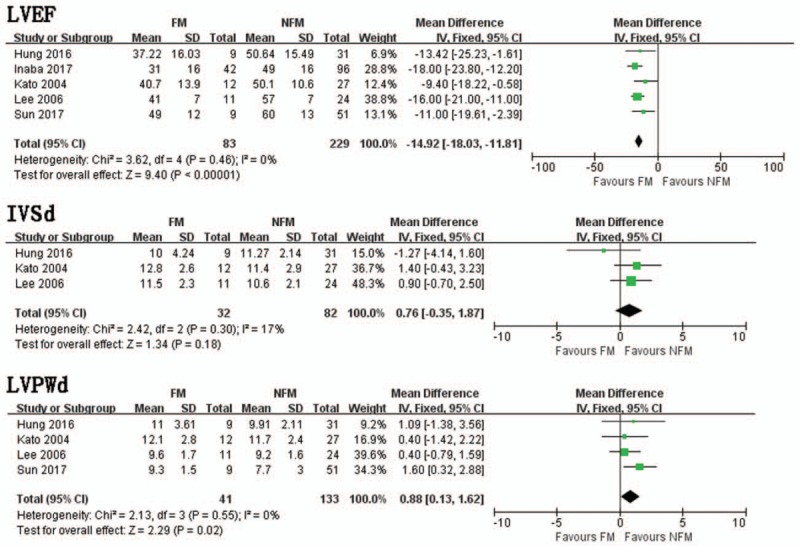
LVEF. Mean difference between FM and NFM; IVSd. Mean difference between FM and NFM; LVPWd. Mean difference between FM and NFM. FM = fulminant myocarditis, IVSd = interventricular septum diameter, LVEF = left ventricular ejection fraction, NFM = non-fulminant myocarditis,

Pooled data from 5 trials demonstrated that FM group had significantly lower LVEF than NFM groups (MD = −14.92, 95% CI −18.03, −11.81, *I*^2^ = 0%, *P *= .46) (Fig. 5). In a pooled analysis of 3 trials, there was no significant difference in LVSd between FM and NFM groups (MD = .76, 95% CI −0.35, 1.87, *I*^2^ = 17%, *P* = .30) (Fig. 5). Four trials studied the distinction in LVPWd between FM and NFM groups. FM group had significantly thicker LVPWd than NFM group (MD = 0.88, 95% CI 0.13, 1.62, *I*^2^ = 0%, *P* = .55) (Fig. 5).

In a pooled analysis of 5 trials, there was no significant difference in LVEDd between FM and NFM groups (MD = −2.75, 95% CI −6.21, 0.72, *I*^2^ = 77%, *P* = .001).

## 4 Discussion

In terms of clinical presentations, we found that syncope and low SBP were early characteristics of FM. Patients with NFM often had chest pain. Others symptoms such as HR, fever, and dyspnea did not have specific diagnostic value. Early cardiac insufficiency may lead to adjacent organ hypoperfusion. Hemodynamic changes that appear early indicated more severe clinical symptoms and more severe heart failure.

Both CK and CK-MB level can be used to evaluate the extent of myocardial cell injury. In this regard, CK-MB level was more specific than the CK level.^[22]^ Troponin levels were more specific for the degree of myocardial damage then CK and CK-MB.^[23]^ When comparing biochemical markers, high CK level was an early characteristic of FM. We did not perform analysis of the MB fraction of creatinine kinase (CK-MB) or troponin-I because of lack of data. Acute myocardial infarction patients with left heart failure often had a wide range of myocardial damage, and peak values of troponin were very high. However, from the data we collected for the FM group, we found that, despite the rises in CK, CK-MB, and troponin-I, the mechanism of severe heart failure could not be explained using damage of myocardial cells alone. BNP and other factors such as ST2 protein predicted worse prognosis not only in heart failure patients,^[24]^ but also in patients supported by implantable cardioverter defibrillators and CRTd devices.^[25,26]^ Patients with FM often died of severe heart failure soon after onset. Therefore, biomarkers of heart failure may play an important role in the diagnosis of FM. Unfortunately these most recent indicators were not abstracted from included studies, and only 2 trials^[15,16]^ reported the relationship between BNP and FM patients. Therefore, we did not make the analysis for lack of data. Furthermore, we found no significance in the level of CRP.

Prolonged QRS duration, ST depression were early characteristics of FM according to our analysis. Widened QRS complexes may reflect that the fact that ventricular depolarizations were prolonged.^[27]^ On the other hand, the phenomenon could represent the extent of cardiac cell injury.^[28]^ Four trials^[16–19]^ showed significant QRS width, presumed to reflect involvement of the conduction system in the extensive myocardial damage. Among the included studies, Ammirati et al^[21]^ reported 9 patients with bundle branch block, 8 of whom developed into FM. Kato et al^[20]^ reported 16 patients had bundle branch block, 9 of whom developed into FM. They concluded that intra-ventricular conduction disturbance was the independent risk factor. Sun et al^[19]^ reported 10 patients with QRS duration >120 ms, 7 of whom developed into FM. Therefore, we should pay more attention to QRS duration in myocarditis patients. VT/VF is thought to be as an early characteristic of FM according to our analysis. Although ventricular arrhythmia is more common in heart failure patients with reduced EF,^[29]^ patients with normal LVEF may present higher ventricular arrhythmic burden if concomitant to a metabolic pro-inflammatory/oxidative status.^[30]^ Hung et al^[18]^ also reported that prolongation of the QTc interval was a relevant factor in FM. Sun et al^[19]^ reported that the prolongation of PR interval was the relevant factor in FM. However, we did not perform the analysis because of lack of more data. From included studies, only Kato et al^[20]^ reported 4 patients had atrial fibrillation, 1 of whom developed into FM. Others did not mention the arrhythmic atrial events or strokes events. The proportion in which atrial arrhythmia caused hemodynamic disorder was far less than that of ventricular arrhythmia. Its impact on clinical outcomes as strokes events usually did not happen when the duration of atrial fibrillation was less than 48 hours.^[31]^ All patients from the included studies were monitored for arrhythmia using electrocardiograph monitoring until discharge or death. The importance of telemonitoring in predicting heart failure hospitalization has been reported.^[32]^ Atrial fibrillation and its high risk of stroke may have long-term effects on patients with myocarditis, including autonomic dysfunction.^[33]^ However, no follow-up or subsequent report have been conducted.

In terms of echocardiographic features, reduced EF and thicker LVPWd were found to be early characteristics of FM. However, we could not find significant changes in LVEDd or IVSd. A echocardiographic study of acute myocarditis found that systolic dysfunction, regional wall motion abnormalities and increased interventricular septal thickness were frequently observed in patients with acute FM.^[34]^ Echocardiographic parameters we collected showed early stage alterations that could not reflect myocardial edema at various stages without a series of follow-up examinations. Currently, cardiac magnetic resonance (CMR) has become the primary noninvasive tool for the diagnosis and evaluation of myocarditis.^[35]^ It provides good accuracy in measurement of LVEF, LVEDd, LVPWd, and IVSd.^[36]^ We should focus on differences between FM and NFM using CMR in further studies.

Although the basic characteristics of patients were not described in detail in the included literature. The past medical history must have some influence on the progress and prognosis of myocarditis. Sardu et al^[37]^ showed that metabolic pro-inflammatory/oxidative factors may affect the prognosis in patients with depressed LVEF. Liao et al^[38]^ reported that damage of renal function renal may affect the prognosis in FM patients with ECMO implanted. Further studies are needed.

FM is an important cause of cardiovascular morbidity and mortality in both children and adults.^[39]^ Adult myocarditis has a wide range of manifestations, ranging from mild asymptomatic illness to acute fulminant disease and even death. The diagnosis of myocarditis is relatively difficult as a result of the various clinical manifestations. The sample sizes of previous single studies were all too small for this rare disease and their results could have been influenced by sample size. Our reason for performing a meta-analysis was to identify the early clinical symptoms and laboratory findings in FM vs NFM. To the best of our knowledge, no one has presented this kind of meta-analysis before.

## 5 Study limitations

Some limitations of this study need to be noted. All the data we collected were from retrospective studies. The population of these studies were mainly from Asia, especially Japan. The results could be influenced by the regional distribution and race, and the funnel plots indicated significant publication bias in some aspects. FM is a rare disease, despite the fact that we included 7 studies (158 FM), the sample sizes remained small.

## 6 Conclusion

We found that the lower SBP, higher CK, wider QRS duration, lower LVEF, thicker LVPWd, higher incidence of ST depression, Vt/Vf and syncope as well as lower incidence of chest pain were early characteristics of FM.

## Author contributions

**Conceptualization:** Zicheng Wang, Da Gao.

**Data curation:** Shengjie Wang.

**Formal analysis:** Shengjie Wang.

**Investigation:** Shengjie Wang.

**Methodology:** Shengjie Wang.

**Project administration:** Haiyan Lin.

**Resources:** Haiyan Lin.

**Software:** Haiyan Lin, Xianlei Cai.

**Supervision:** Yanwei Wang, Xianlei Cai.

**Validation:** Yanwei Wang, Xianlei Cai.

**Visualization:** Yanwei Wang.

**Writing – original draft:** Zicheng Wang.

**Writing – review & editing:** Da Gao.
